# Dedifferentiated proximal tubular epithelial cells in diabetic kidney disease: pathogenic roles, repair potential, and therapeutic implications

**DOI:** 10.3389/fendo.2026.1965334

**Published:** 2026-09-15

**Authors:** Lei Chen, Fei Wang, Tianyi Lv, Lin Liao

**Affiliations:** 1 School of Clinical Medicine, Shandong Second Medical University, Weifang, China; 2 Department of Endocrinology and Metabology, Shandong Key Laboratory of Rheumatic Diseaseand Translational Medicine, The First Affiliated Hospital of Shandong First Medical University & Shandong Provincial Qianfoshan Hospital, Shandong Institute of Nephrology, Jinan, China

**Keywords:** dedifferentiation, diabetic kidney disease, proximal tubular epithelial cell, redifferentiation, tubular repair

## Abstract

Proximal tubular epithelial cells (PTECs) are more susceptible to damage due to their high energy requirements. The apoptosis of PTECs has been thought as the main cause of diabetic kidney disease (DKD). However, the reported apoptotic rate was around 5%-25%, suggesting that apoptosis alone cannot fully explain disease progression. An important question is arised: what happens to these surviving PTECs? Studies showed that many surviving PTECs lost their mature phenotype and entered a dedifferentiated, scattered tubular cell-like state. This adaptive change may offer some protective benefit. However, chronic persistent high-glucose stimulates those dedifferentiated PTECs (dPTECs) to secrete proinflammatory or profibrotic factors and drove tubulointerstitial fibrosis, even renal failure. Therefore, PTECs are not only the “victim”, but also the “driver” in the development of DKD. Current therapeutic strategies, including SGLT2 inhibitors, gut microbiota modulation, and traditional Chinese medicine, may improve the pathological microenvironment associated with dPTECs, but direct evidence that they can reverse PTEC dedifferentiation remains limited. This review summarizes the characteristics and regulatory mechanisms of dPTECs in DKD, discusses their pathogenic roles, heterogeneity, repair potential, and therapeutic relevance, aiming to provide new perspectives for redirecting maladaptive tubular remodeling toward renal repair.

## 1 Introduction

Diabetic kidney disease (DKD) represents one of the most prevalent and severe microvascular complications of diabetes mellitus (1). Proximal tubular epithelial cells (PTECs) undertake crucial physiological functions such as reabsorption and substances transport to maintain renal homeostasis. Because of their special anatomical location, high metabolic activity and susceptibility to damage, PTECs become the primary victims to diabetic injury (2). Recent evidence has increasingly shown that tubular injury is not only a consequence of DKD, but may also contribute to disease progression (2). PTECs apoptosis has long been regarded as an important cause of early DKD and the hot point of research. However, the reported apoptotic rate was only about 5%-25% (3, 4), suggesting that cell death alone could not fully explain the disease progression. This raises an important but often overlooked question: what happens to the 95%-75% surviving PTECs? Recent studies have confirmed that high-glucose stimulation can drive significant phenotypic and functional remodeling of PTECs through multiple pathways. One of the most representative events is dedifferentiation (5). Dedifferentiation is supposed to be an adaptive and transient process in tissue repair. However under chronic sustained stimulation of high-glucose, the reparative program may become maladaptive (6). The dedifferentiated PTECs (dPTECs) may secrete many proinflammatory or profibrotic cytokines and drive to tubulointerstitial fibrosis (7), even renal dysfunction (6). Therefore, the key question is not only whether PTECs survive, but also the direction of dPTECs’ development, improvement or worse.

Currently, the pathogenic roles, heterogeneity, and repair potential of dPTECs in DKD have not been systematically summarized. Although related interventions of sodium–glucose cotransporter 2 inhibitors (SGLT2i), traditional Chinese medicine, and gut microbiota have been reported to improve metabolism, inflammation, and fibrosis in chronic kidney disease (8–10), direct evidence that they can affect PTECs dedifferentiation or redifferentiation in DKD remains limited. This review focuses on the source, regulatory mechanisms, pathogenic roles and repair potential of dPTECs in DKD, and discusses their potential therapeutic value and challenges.

## 2 Definition and characteristics of dPTECs

The concept of dedifferentiation was first proposed on morphology in 1917 to describe the process by which mature cells revert to a less specialized state in response to injury (11). In 1998, Ichimura et al. provided molecular-level evidence of dedifferentiation in acute injury by identifying kidney injury molecule-1 (KIM-1) as the marker for surviving and regenerating epithelial cells (12). In 2014, Berger et al. further demonstrated that tubular regeneration was largely mediated by surviving tubular epithelial cells, which transiently acquired a dedifferentiated, scattered tubular cell-like phenotype (13). In 2020, Su et al. extended the concept to DKD (5). This finding suggests that dedifferentiation is not restricted to acute repair, but may also participate in chronic progression of DKD.

In this context, scattered tubular cells (STCs) are a kind of transitional type cells rather than fixed progenitor cells and often regarded as a classical representation of dPTECs. Phenotypically, dPTECs/STCs are characterized by three major features: loss of mature tubular epithelial identity, reactivation of progenitor-like or developmental programs, and induction of injury-associated molecules such as kidney injury molecule-1 (KIM-1) (14). Some dPTECs may also acquire mesenchymal-like traits, including reduced E-cadherin and increased vimentin expression. But this state should not be simply equated with complete epithelial-to-mesenchymal transition. Notably, dPTECs should be understood as injury-responsive tubular cells located between adaptive repair and maladaptive repair. Under appropriate repair conditions, dPTECs may redifferentiate into functional PTECs, thereby contributing to adaptive repair (15, 16).

## 3 Mechanisms underlying PTECs dedifferentiation in DKD

### 3.1 Core signaling pathways

The transforming growth factor-β (TGF-β)/Smad signaling pathway is one of the central pathway of PTECs dedifferentiation (17). After binding to type II/I transmembrane receptors, TGF-β activates the downstream Smad proteins (mainly Smad2/3) and induces them translocation into the nucleus (**Figure 1**). This process directly regulates the transcription of genes governing cellular proliferation, apoptosis and extracellular matrix remodeling. Sustained activation of this pathway under high-glucose conditiona suppressed the expressions of differentiation-associated genes in PTECs at transcriptional and post-translational levels, and drove the cells dedifferentiation (17).

**Figure 1:**
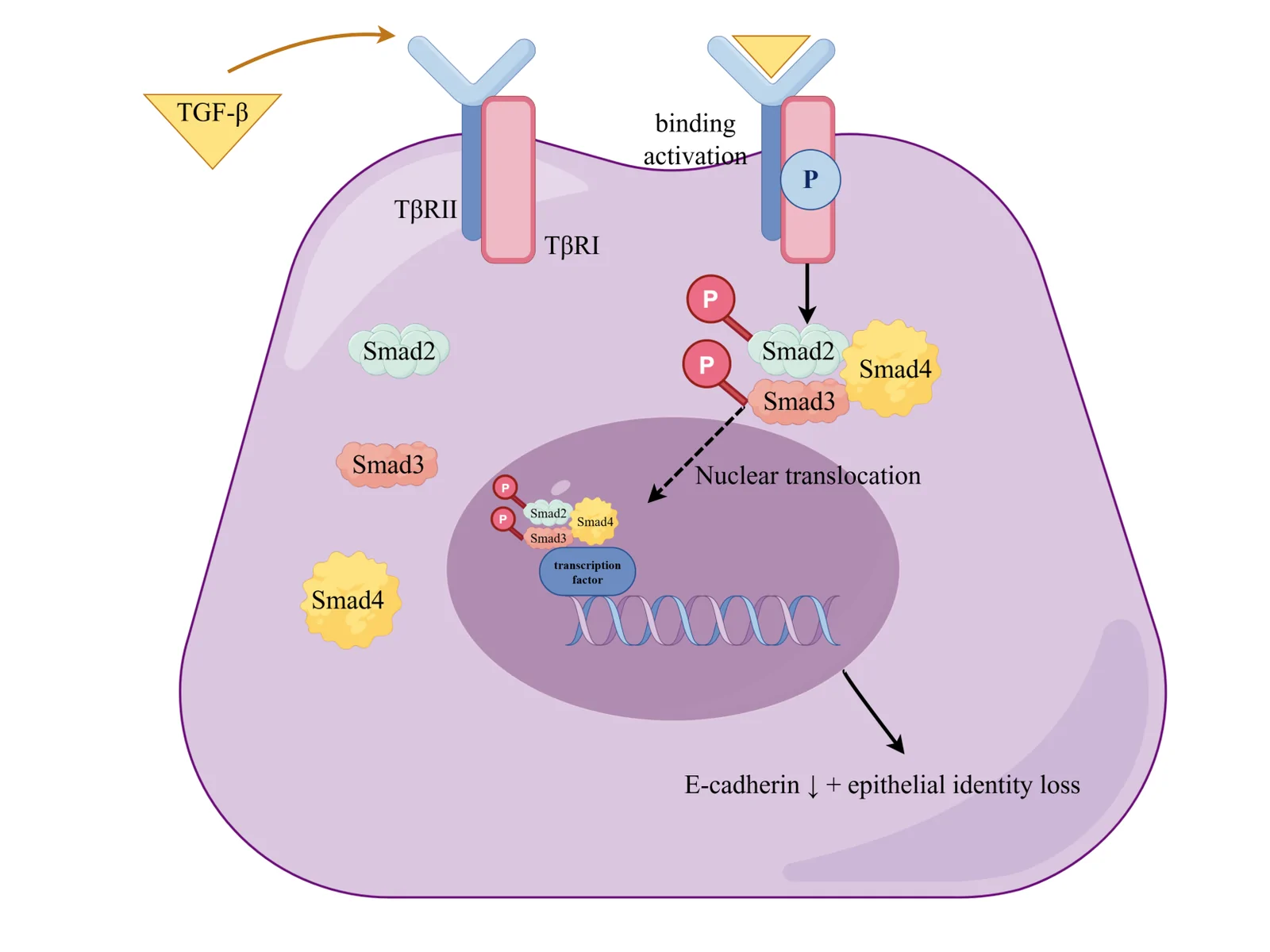
TGF-β/Smad signaling promotes PTECs dedifferentiation. TGF-β binds to TβRII and activates TβRI, leading to Smad2/3 phosphorylation. Phosphorylated Smad2/3 forms a complex with Smad4 and translocates into the nucleus, resulting in cell dedifferentiation. TGF-β, transforming growth factor-β; TβRI, transforming growth factor-β receptor type I; TβRII, transforming growth factor-β receptor type II; Smad, SMAD family member; p-Smad, phosphorylated Smad.

Meanwhile, Wnt/β-catenin signaling pathway is activated continuously in DKD (18). In healthy adult kidneys, the canonical pathway activity is confined to the papilla. When injured, pathway activation occurs in the cortical and medullary regions (19). Evidence from renal injury models suggested that macrophages in the injury microenvironment secreted Wnt ligands such as Wnt7B, which led to the accumulation of β-catenin in nucleus and induced the expressions of transcription factors such as Snail and Twist (20). Interestingly, TGF-β can upregulate Wnt ligand expression and enhance β-catenin stability (21), while activated β-catenin may cooperate with Smad proteins to amplify the transcription of fibrosis genes (22).

In DKD, aberrant Notch signaling pathway is associated with metabolic reprogramming, mitochondrial dysfunction, epithelial-mesenchymal transition and dedifferentiation (23). In addition, high-glucose-treated tubular cells activated the Notch signaling pathway in macrophages through Dll4-containing exosomes (24). This process enhances the inflammatory effect and promotes pathological extracellular matrix remodeling, forming a vicious circle of “dedifferentiation-inflammation-fibrosis”.

### 3.2 Metabolic reprogramming

#### 3.2.1 Mitochondrial dysfunction

Persistent high-glucose environment and inflammation can induce dynamin-related protein 1 phosphorylation at Ser616, promote mitochondrial fragmentation and impaire oxidative phosphorylation (25, 26). This triggers excessive reactive oxygen species (ROS) production and reduce adenosine triphosphate generation, resulting in a cellular bioenergetic crisis. ROS overproduction can further activate injury-related signaling pathways, including TGF-β/Smad and Notch signaling (27). Concurrently, compensatory glycolysis may generate a “Warburg-like” phenotype with increased glucose uptake and lactate accumulation. Lactate may reinforce TGF-β signaling and acidify the extracellular milieu that suppresses histone deacetylase activity and the expression of epithelial markers, thereby promoting PTECs dedifferentiation (28, 29). Therefore, mitochondrial dysfunction is not merely a consequence of diabetic injury, but may serve as an upstream driver linking metabolic stress to maladaptive tubular remodeling.

#### 3.2.2 Inactivation of metabolic sensors

PTECs rely on metabolic signaling axes to coordinate nutrient sensing, lysosomal degradation and proliferative differentiation for renal homeostasis (30). Under the stress of high-glucose and lipid toxicity, key sensors such as adenosine monophosphate-activated protein kinase (AMPK) and NAD-dependent deacetylase sirtuin 1 (SIRT1) become inactivated, which diminishes their ability to maintain energy balance and ultimately lead to the loss of epithelial phenotype (31). Furthermore, downregulation of SIRT1 may disturb forkhead box O-related stress responses and exacerbate tubular dysfunction (32, 33). Thus, dysfunction of metabolic sensors not only reshapes cellular metabolism, but also removes the “brakes” on inflammation, oxidative stress, and fibrosis. This metabolic failure is a key pathological mechanism linking PTECs dedifferentiation to tubulointerstitial fibrosis in DKD.

### 3.3 Podocyte-derived exosomes

The expression of podocyte-derived exosomes microRNA-221 (miR-221) was significantly upregulated under high-glucose (5). After being transferred to PTECs, miR-221 directly inhibited the expression of Dickkopf-2, which relieved its inhibition on Wnt/β-catenin pathway, led to β-catenin Tyr-654 phosphorylation, nuclear translocation, PTECs dedifferentiation and functional impairment (5) (**Figure 2**). Animal study showed that miR-221 inhibitor reduced dedifferentiation-associated protein expression and alleviated tubular injury (34). These findings suggest that miR-221 acts as an important mediator of glomerular–tubular crosstalk and may become a novel therapeutic target for DKD.

**Figure 2:**
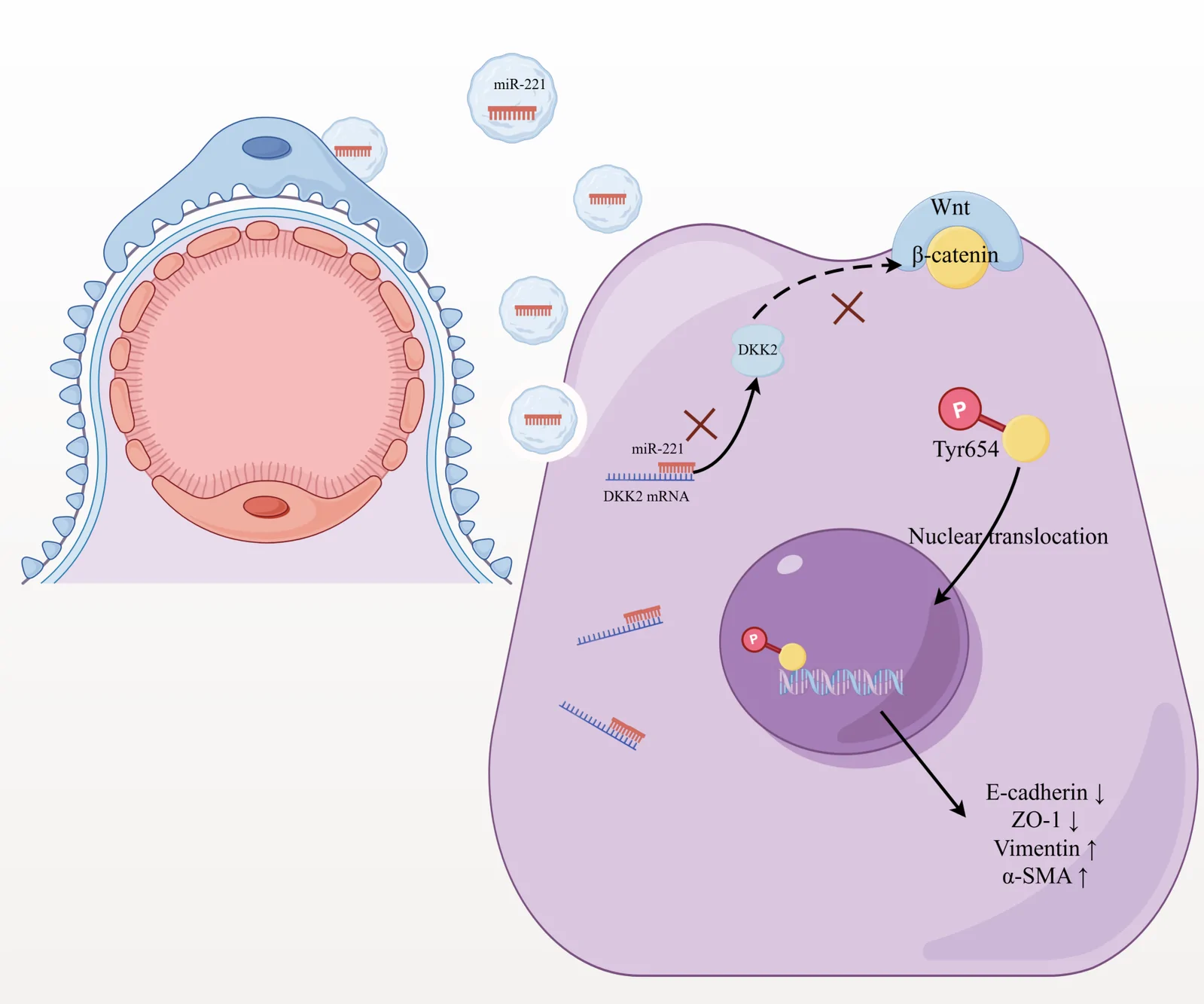
Podocyte-derived extracellular vesicles promote PTECs dedifferentiation through the miR-221/DKK2/β-catenin axis. Podocyte-derived EVs transfer miR-221 into PTECs, where miR-221 suppresses DKK2 and leads β-catenin phosphorylation. Subsequent β-catenin nuclear translocation is associated with acquisition of a dedifferentiated phenotype. EVs, extracellular vesicles; miR-221, microRNA-221; DKK2, Dickkopf WNT signaling pathway inhibitor 2; PTECs, proximal tubular epithelial cells; β-catenin, beta-catenin; ZO-1, zonula occludens-1; α-SMA, alpha-smooth muscle actin.

### 3.4 Aberrant DNA methylation

In DKD, PTECs’ function-related genes exhibit differential methylation at specific CpG sites. The genes related to gluconeogenesis and reabsorption are hypermethylated, while the genes related to oxidative stress and cytoskeletal remodeling tend to be hypomethylated (35). These methylation changes may impair tubular metabolic and transport functions or enhance stress responses, thereby promoting the loss of mature epithelial identity. Metabolic genes regulated by hepatocyte nuclear factor 4 alpha displayed demethylation in healthy kidneys, a process absent in DKD (36). Moreover, activation of DNA methyltransferase 1 in PTECs silenced anti-fibrotic genes and aggravated the phenotypic loss (37). Together, aberrant DNA methylation may link metabolic dysfunction, stress responses, and PTEC dedifferentiation in DKD.

## 4 Potential pathogenic roles of dPTECs in DKD

In acute injury, PTECs recover function and morphology through programmed repairment of “dedifferentiation-proliferation-redifferentiation” (15). However, dPTECs are often “locked” in a maladaptive phenotype under sustained high-glucose, oxidative stress and inflammation, which makes it difficult to redifferentiate and restore epithelial homeostasis (38). This continuously activates the pathological pathway of tubulointerstitial fibrosis and renal dysfunction.

### 4.1 Loss and deterioration of renal tubular function

Tubulopathy precedes glomerulopathy in DKD. Significant tubular damage was observable even in non-albuminuric DKD (39), and has been proven to be a key driver of renal dysfunction (40). dPTECs lose polarity, disrupt the brush border and downregulate the critical transporters, causing abnormal reabsorption of glucose, amino acids and electrolytes. When dPTECs fail to redifferentiate, tubular reabsorptive dysfunction may persist. This functional abnormality can not only disrupt renal metabolic homeostasis but also impair tubuloglomerular feedback, thereby further promoting the progression of DKD (2).

### 4.2 Driving inflammation and promoting tubulointerstitial fibrosis

PTECs are not just passive victims of renal fibrosis, but also interact with immune cells and interstitial cells by inflammatory cytokines, chemokines, and profibrotic factors (41, 42). Eymael et al. found that the abundance of dPTECs was positively correlated with the degree of renal interstitial fibrosis. Transcriptomic analysis further revealed nuclear factor kappa B, tumor necrosis factor-α and various inflammation-related pathways were significantly up-regulated in these cells (43). These findings suggested that dPTECs was not a simple loss of function, but might also be related to maladaptive inflammatory responses. In studies of DKD, abnormal PTEC subsets of VCAM1^+^ or KIM-1^+^ have been reported to exhibit cellular communication characteristics (44). These distinct cell populations can secrete a range of cytokines and chemokines, contributing to macrophage recruitment, lymphocyte infiltration and myofibroblast activation (**Figure 3**).

**Figure 3:**
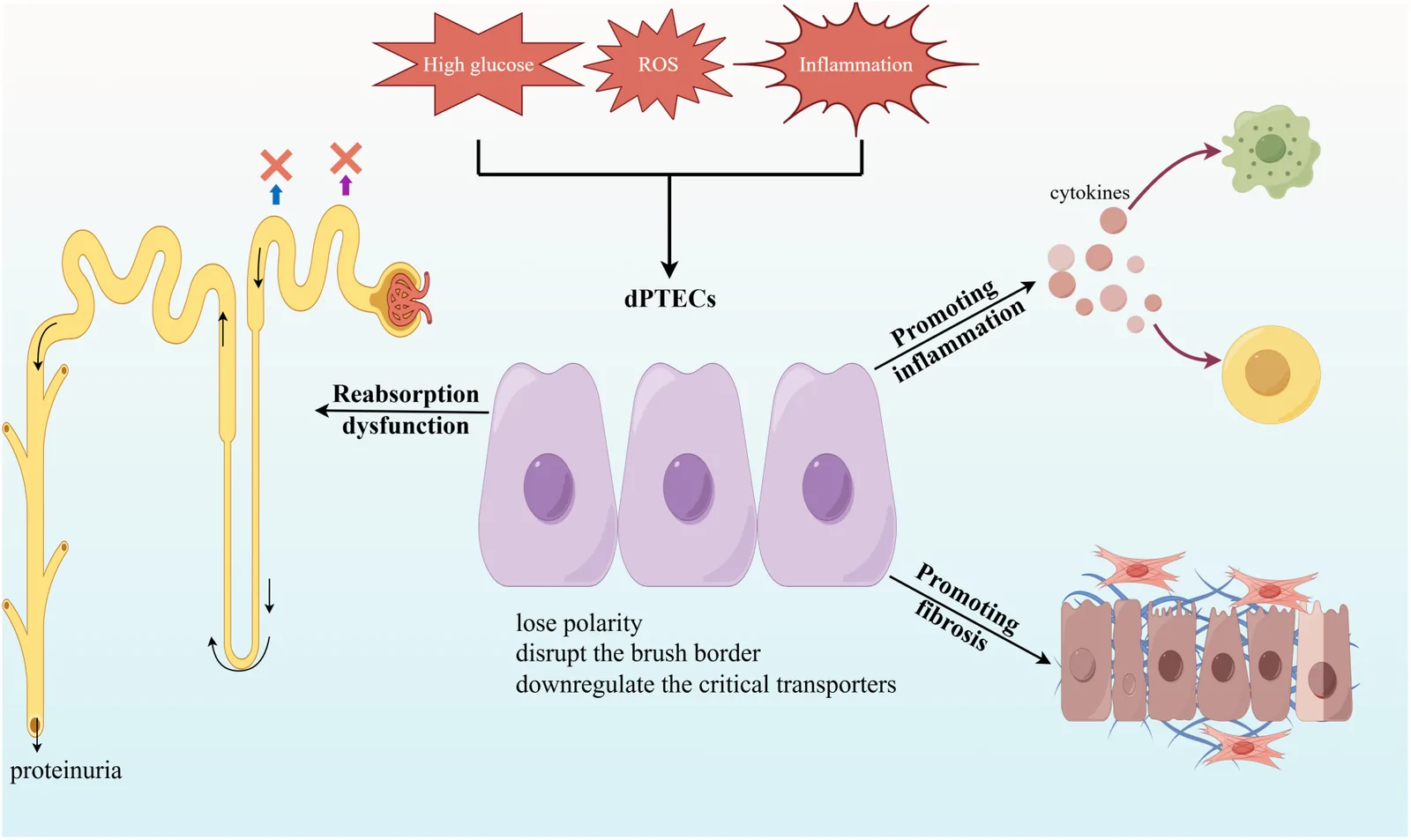
Pathogenic roles of dPTECs in diabetic kidney disease. Persistent exposure to stimuli such as high glucose, oxidative stress and inflammation promotes the maintenance of PTECs in a dedifferentiated state. dPTECs lose cell polarity and brush-border integrity, and exhibit reduced expression of key transporters, resulting in impaired tubular reabsorption. In addition, dPTECs can recruit and activate macrophages, lymphocytes, and fibroblasts, thereby driving inflammatory progression and tubulointerstitial fibrosis. ROS, reactive oxygen species.

Taken together, current evidences support that the existing dPTECs exhibit both pro-inflammatory and pro-fibrotic phenotypes, participate in immune cell recruitment and interstitial cell activation, driving inflammation and promote renal interstitial fibrosis. However, in the context of DKD, there is still a lack of causal evidence on which mechanism dPTECs directly promote disease progression, which is also a question that needs further verification.

## 5 Repair potential and challenges of dPTECs in DKD

In acute kidney injury, dPTECs exhibit strong regenerative potential, capable of proliferating and redifferentiating into functional tubular cells. Thus, they are considered a crucial cellular source for repair (13). In DKD, transcriptomic data and biopsies of human kidney samples have revealed a significant increase in dPTECs, especially the SOX9^+^ cells with better reparative capacity (45). Nevertheless, how to redirect dPTECs toward functional redifferentiation remains a big challenge.

### 5.1 Lack lineage tracing and dynamic change validation

Current evidences on dPTECs in DKD come from immunostaining and transcriptomic data, indicating that dPTECs may derive from surviving PTECs that acquire a dedifferentiated phenotype (5, 43). However, these evidences lack lineage tracing to confirm precise origin and dynamic changes, especially the latter. How to make dPTECs redifferentiate is our future work direction and focus.

### 5.2 Heterogeneity of dPTECs

The dPTECs display substantial heterogeneity, with distinct subsets contributing differently to tubular repair or fibrosis (43). The SOX9^+^ subset is characterized by its strong regenerative potential (45), whereas the CD24+/CD133^+^ subset exhibits stem-like properties but may produce maladaptive signals under inflammation (46). In contrast, the Vimentin^+^ subset represents a dedifferentiated state; however, direct evidence supporting its redifferentiation into functional PTECs remains limited (15). This heterogeneity indicates the uncertainty of dPTECs, which underscores the need for refined subset.

### 5.3 Effect of hostile diabetic milieu

Unlike acute injury, DKD is a chronic disease characterized by persistent metabolic stress, including hyperglycemia, oxidative stress and low-grade inflammation. These pathogenic factors damage PTECs, tend to maintain their dedifferentiated phenotype, inhibit the redifferentiation of dPTECs and finally lead to repair failure (47). Therefore, improving the metabolic and inflammatory microenvironment may create more favorable conditions for dPTECs to regain mature tubular identity.

However, the failure of redifferention cannot be fully explained by metabolic stress and inflammation alone. Clinical observation showed that even patients with well-controlled glucose still developed progressive DKD, suggesting that intrinsic cellular programs may also maintain the dedifferentiated state (48). Therefore, identifying molecular mechanisms that influence the regenerative potential of dPTECs represents an important direction for future research.

### 5.4 Technical bottlenecks in cell therapy

Damaged kidneys release specific homing signals to attract endogenous dPTECs for repair (49, 50), so researchers have proposed innovative ideas for exogenous dPTECs therapy. This highly promising strategy faces multiple challenges. First, dPTECs account for extremely low proportion of kidney cells, and invasive renal puncture limits the feasibility. Second, how to stably amplify dPTECs *in vitro* and maintain their regenerative phenotype remains unresolved. Furthermore, mature protocols for transplanted cell homing, integration, and functional recovery are lacking. Finally, dedifferentiated cells carry risks of abnormal proliferation, so its safety needs furth improvement.

## 6 Current treatments and their potential effects on dPTECs

Currently, the management of DKD has changed from glucose control alone to a comprehensive treatment for hemodynamic, metabolic disorder, inflammation and fibrosis (1). SGLT2i is a hypoglycemic drug with definite renal protection (8). SGLT2i can alleviate glomerular hyperfiltration and reduce proteinuria by restoring tubuloglomerular feedback (2, 8). In addition, SGLT2i exerts anti-inflammatory and anti-fibrotic effects. Studies have shown that SGLT2i can suppress the expression of inflammatory mediators such as MCP-1 and attenuate renal inflammation (51). Dapagliflozin can delay renal fibrosis in DKD by inhibiting YAP/TAZ signaling (52). These effects of SGLT2i may create more favorable microenvironment for dPTECs to regain a mature phenotype. However, direct evidence that SGLT2i can reverse dPTEC dedifferentiation is still lacking.

In addition to SGLT2i, gut microbiota and traditional Chinese medicine are also important directions of DKD treatment research in recent years. gut microbiota imbalance can impair intestinal barrier integrity and promote renal inflammation and fibrosis through mechanisms involving microbiota-derived metabolites and systemic inflammation (53). Therefore, modulation of the gut microbiota is expected to improve both the systemic and local renal microenvironment, and indirectly alleviated PTEC injury. Traditional Chinese medicine may improve metabolic dysfunction, oxidative stress, inflammation and fibrosis through multiple mechanisms. It has been shown that astragaloside IV inhibits the IRE1α/NF-κB/NLRP3 inflammatory axis and reduces endoplasmic oxidative stress and inflammatory responses in PTECs (54). Salvia miltiorrhiza and its main active components tanshinone and salvianolic acid can protect PTECs by regulating Nrf2/Keap1, TGF-β/Smad and NF-κB pathways (55, 56). It is worth noting that these pathways overlap with the dedifferentiation mechanism of PTECs, which suggests that traditional Chinese medicine therapy may have the potential to regulate the state of dPTECs.

Overall, these treatments improve pathologies associated with the presence of dPTECs and possibly indirectly affect the status of dPTECs. However, relevant studies often take renal function, inflammatory indicators or fibrosis degree as the primary endpoints, and the direct evidence for the phenotypic transition of PTECs is still very limited. This is a research direction worthy of attention, and may also provide new perspective for DKD.

## 7 Conclusion

PTECs dedifferentiation may represent an important process linking tubular injury, failed repair, and DKD progression. Persistent dPTECs exhibit functional impairment and may be involved in inflammation and fibrosis. At the same time, dPTECs still retain a certain repair potential. Although current therapies can improve the associated pathological microenvironment, direct evidence that they can alter the phenotype and function of dPTECs remains limited. Future studies should clarify the dynamic changes and regulatory mechanisms of dPTECs and explore strategies to promote their redifferentiation.
